# Linkage and exome analysis implicate multiple genes in non-syndromic intellectual disability in a large Swedish family

**DOI:** 10.1186/s12920-019-0606-4

**Published:** 2019-11-06

**Authors:** Eva Lindholm Carlström, Jonatan Halvardson, Mitra Etemadikhah, Lennart Wetterberg, Karl-Henrik Gustavson, Lars Feuk

**Affiliations:** 1 Department of Immunology, Genetics and Pathology, Science for Life Laboratory Uppsala, Uppsala University, Box 815, SE-751 08 Uppsala, Sweden; 2 Department of Clinical Neuroscience (CNS), K8, Karolinska Institutet, Stockholm, Sweden

**Keywords:** Affymetrix genome-wide human SNP Array 6.0, Complex disorder, Genome wide analysis, Large pedigree, Sequencing

## Abstract

**Background:**

Non-syndromic intellectual disability is genetically heterogeneous with dominant, recessive and complex forms of inheritance. We have performed detailed genetic studies in a large multi-generational Swedish family, including several members diagnosed with non-syndromic intellectual disability. Linkage analysis was performed on 22 family members, nine affected with mild to moderate intellectual disability and 13 unaffected family members.

**Methods:**

Family members were analyzed with Affymetrix Genome-Wide Human SNP Array 6.0 and the genetic data was used to detect copy number variation and to perform genome wide linkage analysis with the SNP High Throughput Linkage analysis system and the Merlin software. For the exome sequencing, the samples were prepared using the Sure Select Human All Exon Kit (Agilent Technologies, Santa Clara, CA, USA) and sequenced using the Ion Proton™ System. Validation of identified variants was performed with Sanger sequencing.

**Results:**

The linkage analysis results indicate that intellectual disability in this family is genetically heterogeneous, with suggestive linkage found on chromosomes 1q31-q41, 4q32-q35, 6p25 and 14q24-q31 (LOD scores of 2.4, simulated *p*-value of 0.000003 and a simulated genome-wide p-value of 0.06). Exome sequencing was then performed in 14 family members and 7 unrelated individuals from the same region. The analysis of coding variation revealed a pathogenic and candidate variants in different branches of the family. In three patients we find a known homozygous pathogenic mutation in the *Homo sapiens solute carrier family 17 member 5* (*SLC17A5*), causing Salla disease. We also identify a deletion overlapping *KDM3B* and a duplication overlapping *MAP3K4* and *AGPAT4*, both overlapping variants previously reported in developmental disorders.

**Conclusions:**

DNA samples from the large family analyzed in this study were initially collected based on a hypothesis that affected members shared a major genetic risk factor. Our results show that a complex phenotype such as mild intellectual disability in large families from genetically isolated populations may show considerable genetic heterogeneity.

## Background

Intellectual disability (ID) is a common disorder that affects approximately 1% of the population world-wide [1]. The diagnosis is divided into severe, moderate and mild with intelligence quotient (IQ) of less than 35, < 50 and < 70 respectively. ID can also be grouped into syndromic and non-syndromic ID. Patients with syndromic ID have other symptoms in addition to ID [2], while non-syndromic patients do not have any additional correlated clinical features. Males are more often affected than females and genetic studies on the X chromosome have led to the identification of causative variants in more than 100 genes, out of the approximately 750 genes located on the X-chromosome [3, 4]. Out of the 750 X-linked genes the majority cause syndromic ID while 50 have been found for non-syndromic ID. However, mutations in several X-linked genes causing intellectual disability can give rise to both non-syndromic and syndromic forms [5] and it is likely that ID genes on autosomes also contribute to both syndromic and non-syndromic forms.

Although many genes have been identified, the genetic basis of the disorder is still unclear in the majority of patients, especially those with non-syndromic ID [6]. ID is highly heterogeneous and it is estimated that more than 2500 autosomal ID genes exist. Sporadic ID in non-consanguineous families are primarily caused by de novo mutations, while in populations with high levels of consanguinity, most ID-causing mutations are recessive [7].

While severe ID is often caused by variants in one single gene, it is believed that the genetic etiology of mild ID is more complex, with multiple genes involved. Moreover, environmental factors such as infection and chemical agents may perturb brain development in the fetus, resulting in ID [2]. Therefore, when elucidating causes to heterogenic diseases such as ID, and milder forms of ID specifically, large families could be particularly useful, as the underlying causes are more likely to be genetic and homogenous.

Several studies of ID in large families have previously been reported [8–11]. The majority of the studies included families with consanguineous marriages, which have led to the identification of mutations causing recessive forms of ID. For ID with more complex inheritance, where the disease is affected by oligogenic or polygenic inheritance, larger families are required. Only a limited number of such families are described in literature [12].

The family presented in our study was previously analyzed using low-resolution approaches [13]. However, no linkage region or potential disease-causing CNVs were identified. Since ID clearly segregates in the family, we decided to perform a new study, using a denser probe and marker spacing. The samples were first analyzed using Affymetrix Genome-Wide human SNP array 6.0 for linkage- and CNV analysis. In addition, we performed exome sequencing of a subset of the family members, as well as seven additional unaffected individuals from the same geographical area, to detect disease associated single nucleotide variants (SNVs). Identified SNVs were validated using Sanger sequencing in a larger number of individuals from the family. The results highlight that multiple genes appear to cause ID in the family.

## Methods

### Clinical materials

**Fig. 1:**
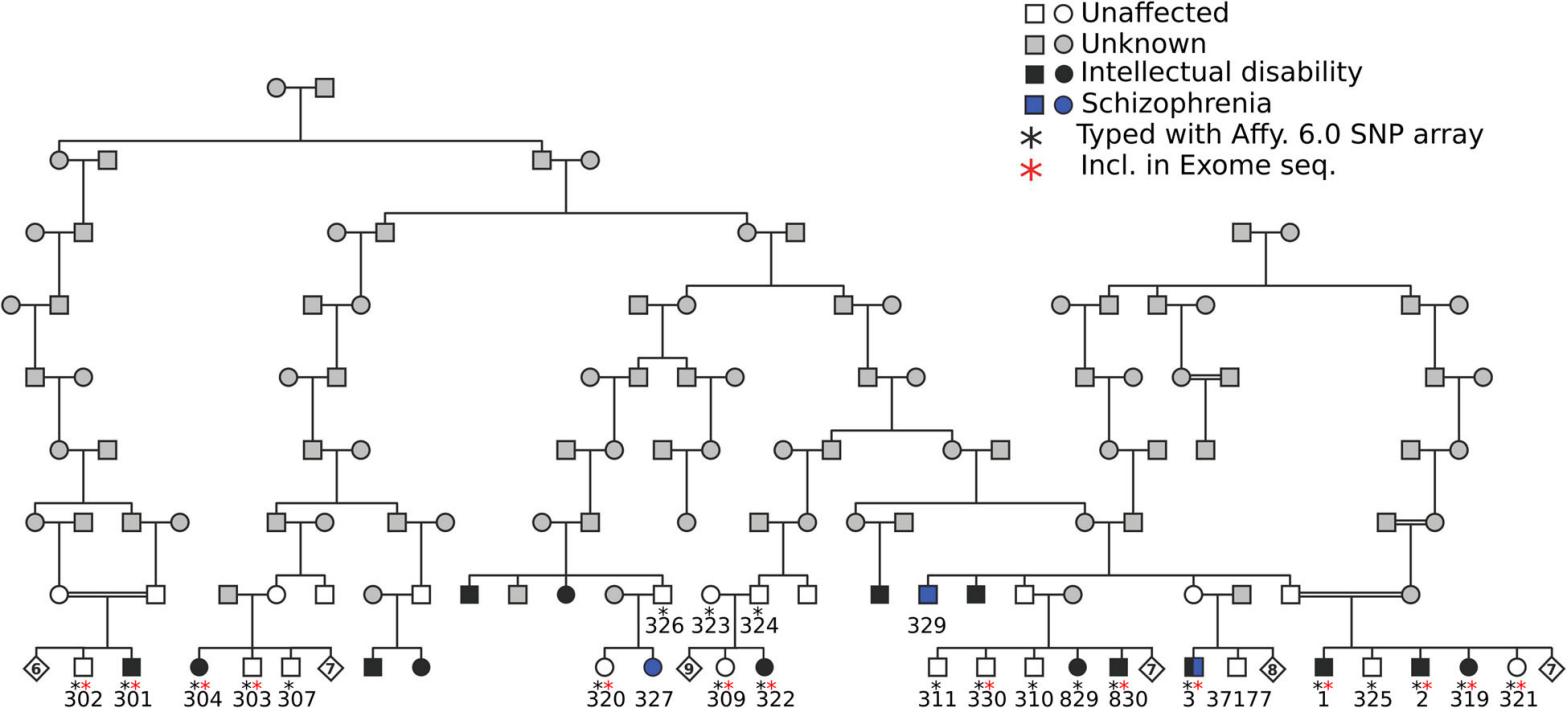
The large multi-generational Swedish family. The diamond symbol with a number indicates additional siblings that are unaffected but from whom we did not have DNA. Black and blue symbols depict individuals diagnosed with ID and schizophrenia, respectively. One individual is diagnosed with both ID and schizophrenia (individual 3). The asterisks indicate the individuals that were included in the linkage analysis and the exome sequencing

The sample set included in our study has been previously described [13, 14]. Briefly, all samples belong to the same pedigree originating from a genetic isolate from Sweden, connected to a common ancestor eight generations past (Fig. 1). Colonists from Northern Finland, many of them related, settled in the Northern part of Sweden close to Finland during the 17th and 18th centuries. Great distances between settlements contributed to the creation of genetic isolates [14]. During the 1950s an epidemiological study was performed in the area that, besides a high prevalence of ID, revealed a high prevalence of schizophrenia. Patients with ID were short with a pyknic body type. Neurological and psychiatric stigmata of various functions and degrees were previously recorded. Neurogenic hearing defects, impaired vision, pathological EEG and widened subarachnoidal space found in computed tomography, were among these stigmata, as described previously [14].

In the present study, nine individuals with intellectual disability and 13 unaffected relatives from the same family were analyzed (Fig. 1). In addition, seven unaffected unrelated individuals from the same geographical area were analyzed. Affected individuals had a mild to moderate intellectual disability with an IQ of 50 or slightly below [14].

### Karyotyping

Karyotyping was performed to exclude large rearrangements as a cause of the disease, using blood samples from one affected individual (Fig. 1, sample no. 3) and an unaffected brother (sample no. 37177). The analysis was performed using standard protocols at the Department of Immunology, Genetics and Pathology, division of Clinical Genetics.

### CNV analysis

CNV calling from the array data was performed with the Genotyping Console™ Software and visualized using Chromosome Analysis Suite (ChAS), provided by Affymetrix. CNVs were selected for further evaluation if they were present in affected individuals only, had a size larger than 10 kilobases and were supported by at least 10 SNPs.

### Linkage analyses

Nine individuals affected with ID and 13 unaffected individuals (indicated with a black asterisk in Fig. 1) were genotyped with the Affymetrix Genome-Wide Human SNP Array 6.0, at the SNP&SEQ Technology Platform at SciLifeLab in Uppsala. The SNP data obtained from the array was used to perform non-parametric multipoint analyses using SNP High Throughput Linkage analysis system (SNP HiTLink) [15] and the Merlin software [16]. From previous analysis we know that the inheritance of ID is complex in the family and the mode of inheritance is unknown [13]. By choosing a non-parametric method (also known as a parameter-free methods) we did not need to specify a disease model. Merlin cannot handle the large family as a single pedigree and we used PedCut 1.19 to divide the pedigree into sub-pedigrees with 24 bit size [17]. With SNPHiTLink we selected SNP markers with maximum linkage disequilibrium (LD) of 0.2 (D’ = 0.2 and r^2^ = 0.2), based on the family we studied, a minimum call rate of 1.0 and Hardy-Weinberg equilibrium of 0.00001. It is important that SNPs used for linkage analysis are not in LD, to reduce the risk of false positive results due to close position of the markers. With SNPHiTLink closely spaced SNPs can easily be removed from the analysis. We ended up with approximately 20,000 SNPs that were not in LD, with high information content, that were used in the linkage analyses. In addition to multipoint analyses we performed single-point and parametric analyses, using both dominant and recessive models. There are also methods to perform digenic inheritance analysis but it is not clear what thresholds for significance that should be used for such methods and their power over single-locus analysis methods is questioned [18].

In an attempt to perform multipoint analysis on the whole pedigree as one family we used Superlink-Online SNP [19] however, the memory required for the computation exceeded the capabilities of the system.

### Exome sequencing

Exome sequencing was performed on eight individuals diagnosed with ID and six unaffected family members (indicated with a red asterisk in Fig. 1). In addition, we analyzed seven unaffected individuals from the genetic isolate, who are not closely related to the affected individuals. The sequencing was performed at Uppsala Genome Center. The samples were prepared using the Sure Select Human All Exon Kit (Agilent Technologies, Santa Clara, CA, USA) and sequenced using the Ion Proton™ System. Reads were mapped to the human reference genome UCSC hg19 using the TMAP 5.0 (Life Technologies) software. Single nucleotide variants (SNVs) and small insertion/deletion variants were detected using Torrent Suite 5.0 (Life Technologies).

In the initial analysis, variants were filtered and kept based on the following criteria: a frequency of less than 0.01 in dbSNP [20], 1000 Genomes Project [21] and ExAC [22]; if present in more than four affected individuals; if located within the four linkage regions; resulting in non-synonymous amino acid changes, stop-gains or affecting splice sites and could be confirmed by Sanger sequencing. In a later step all variants affecting the coding sequence in patients (nonsense, nonsynonymous and splice variants) were screened.

### Sanger sequencing

Primers for Sanger sequencing were designed using the online tool Primer3 [23, 24]. DreamTaq DNA polymerase (Thermo Scientific, #EP0701) was used to amplify the DNA. For regions that could not be amplified with DreamTaq the enzyme Phusion Hot Start II High Fidelity DNA polymerase (Thermo Scientific, F-549S) was used, according to the manufacturer’s instructions. PCR products were purified with QIAquick PCR purification kit (Qiagen 28,106) or QIAquick Gel Extraction kit (Qiagen, # 28704) after gel purification. Sanger sequencing was then performed according to standard protocols. Five SNVs satisfied the initial filtering criteria and were validated with Sanger sequencing in nine affected and 13 unaffected family members. To evaluate the variants further we performed Fisher’s exact test to determine whether the SNVs segregated with affection status. The parents were omitted from the statistical analyses. The variants that were detected in the exome sequencing analysis and validated with Sanger sequencing were submitted to ClinVar (http://www.clinvar.com).

### Evaluation of deleteriousness of identified SNVs

Combined Annotation Dependent Depletion (CADD) scores (more specifically, CADD PHRED scores) were used for scoring of the deleteriousness of identified variants [25]. Mutation taster [26] and the SIFT tool was used to predict if the variants were disease causing and MUPro 1.0 [27] was used to evaluate whether the SNVs affected the protein stability. Genomic Evolutionary Rate Profiling (GERP) scores were used to estimate the conservation of the positions for the identified variants, using the online tool SeattleSeq Annotation 138. The GERP score ranges from − 12.36 to 6.17, with 6.17 being the most conserved.

## Results

Karyotyping was performed for one affected and one unaffected family member. The results showed no large chromosomal rearrangements, neither in the affected patient nor in the unaffected brother (Additional file 1: Figure S1). Affymetrix SNP Array 6.0 was used to analyze 22 individuals (9 affected and 13 controls), and the SNP data was used for CNV detection and linkage analysis. The CNVs analysis was focused on variants that included at least 10 markers and had a size of at least 10 kb. None of the identified CNVs were found to segregate with affection status in the family. However, we did find a small number of CNVs with sizes larger than 10 kb in single affected individuals that were not present in the general population (according to the Database of Genomic Variants [28]) nor in unaffected family members (Table 1). The largest of the identified CNVs was a duplication found in individual 3, which was located on chromosome 6q26 located in a region reported to be involved in patients with ID [29]. The CNV had a size of approximately 200 kb and included the genes *MAP3K4* and *AGPAT4*.

**Table 1:** Summary of the results from the CNV analysis

Chromosome	Start	End	Size (kb)	CNV	Patient	Genes within or close to the CNV
5q31.2	137,660,826	137,724,036	63	del	304	CDC25C, FAM53C, KDM3B
6q26	161,354,191	161,558,170	204	dup	3	MAP3K4, AGPAT4
7q11.22	71,665,416	71,714,246	49	del	1	CALN1
9q31.1	105,763,417	105,822,396	59	del	322	CYLC2
12p13.31	6,643,315	6,671,120	28	dup	830	GAPDH, IFFO1, NOP2
12q24.23	119,981,576	119,994,376	13	del	301	No genes
19p13.11	19,943,909	19,966,901	19	dup	322	No genes
19q13.42	55,622,908	55,641,955	19	dup	830	PPP1R12C

**Fig. 2:**
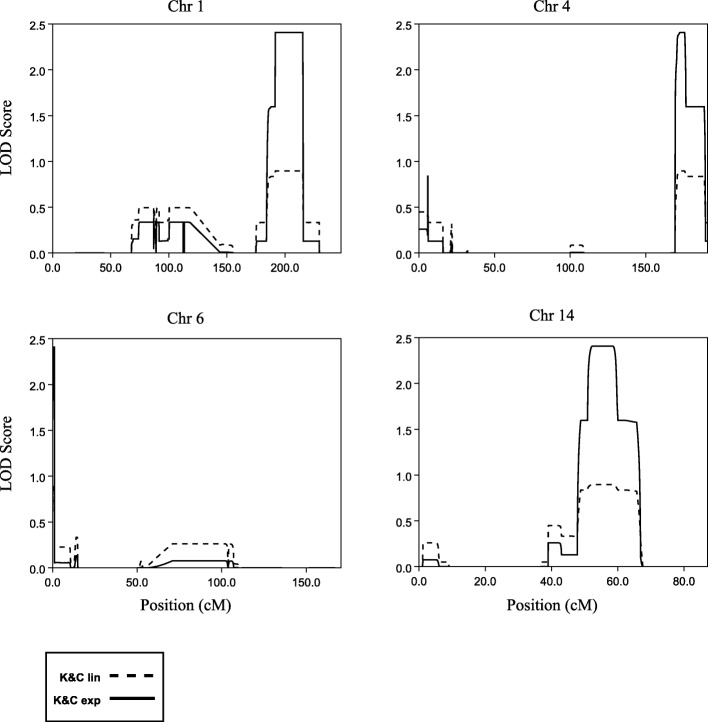
The 4 graphs show the LOD scores for chromosomes 1, 4, 6 and 14 where the maximum LOD score of 2.4 were reached. The dashed and solid lines indicate the LOD values for Kong and Cox linear model (K&C lin) and Kong and Cox exponential model (K&C exp), respectively

Genome-wide multipoint NPL-analysis revealed four regions with LOD scores 2.4, which was the maximum possible LOD score that could be obtained in the analyses, on chromosomes 1q25.3-q41, 4q32-q35, 6p25 and 14q24-q31 (Table 2 and Fig. 2). We also performed parametric LOD score calculations, using both dominant and recessive models, with a penetrance of 0.8 and phenocopy rate of 0.05. None of the two models generated evidence for linkage, supporting the previous findings that ID is inherited in a complex mode in the family [13].

**Table 2:** Summary of the linkage results using data from the Affymetrix 6.0 SNP Array

Linkage to region	Start position	End position	Region size	Max LOD
1q25.3-1q41	184,916,682	216,549,082	32 Mb	2.4
4q32.3-q35.2	169,474,972	189,433,290	20 Mb	2.4
6p25.3	205,947	1,308,575	1 Mb	2.4
14q24.1-14q31.3	68,190,649	86,859,696	19 Mb	2.4

In general, a LOD score of 3 is considered significant. However, since the maximum LOD score that could be obtained in our analyses is 2.4 we wanted to estimate the rate at which a LOD of 2.4 would be reached by chance. Therefore, we performed simulations of 2,000,000 SNPs, corresponding to 100 genome-wide scans with 20,000 markers, approximately the number of markers included in our study, under the assumption of no linkage. Among the 2,000,000 simulated markers, the maximum LOD of 2.4 was only reached six times, which corresponds to a *p*-value of 0.000003. At the genome wide level the p-value is estimated to 0.06, since six out of 100 simulated scans reached a LOD of 2.4 (data not shown).

### Sequencing

In the initial filtering we searched for variants within the linkage regions that were present in at least four patients and missing or present at low frequency in public SNP databases. Using this strict filtering on the SNVs identified by exome sequencing, only five SNVs in three different genes, *Translocated Promoter Region, Nuclear Basket Protein* (*TPR*), *SLIT-ROBO Rho GTPase Activating Protein 2* (*SRGAP2*) and *ACOT4* remained (Table 3). Sanger sequencing was performed to validate the SNVs and Fisher’s exact test was used to evaluate whether the identified variants were associated with ID among the family members. Only the SNV in *TPR* (rs199892357) showed significant association with affection status (*p*-value 0.012, Table 3), while variants in *ACOT4* reached a p-value of 0.058 (Fisher’s exact test).

**Table 3:** Summary of the SNVs identified in the linkage peaks using exome sequencing

Alteration	Gene	Amino acid alteration	CADD PHRED/GERP	*P*-value** (one-tailed)
chr1(GRCh37):g.186329959A > G	*TPR*	p.(Ile346Thr)	23.9/4.47	0.012
chr1(GRCh37):g.206566903G > A	*SRGAP2*	p.(Arg148Hist)	34/5.6	1.0
chr14(GRCh37):g.14_17delinsAATG*	*ACOT4*	p.(Ala189_Tyr190delinsGlnCys)	22/5.25	0.058

Only SNVs identified in at least 4 affected individuals, resulting in missense or loss-of-function, with a frequency less than 0.01 in the general SNV databases are listed

Chr14g.14_17delinsAATG* indicate the alteration g.74060514_74060517delinsAATG on chromosome 14

Transcripts used for the annotations were NM_003292 (*TPR*), NM_001271872 (*SRGAP2*), and NM_152331 (*ACOT4*)

** *P*-values are not corrected for the three tests that were performed

*TPR* is coded on chromosome 1 and contains 51 exons. The identified SNV results in the non-synonymous amino acid change p.(Ile346Thr), located in the tenth exon, between the NPC association- and HSF1 interaction domains. Amino acid 346 is conserved from human to *Xenopus Tropicalis* (UCSC genome Browser, http://genome.ucsc.edu). The variant is predicted to be disease causing according to MutationTaster [26], but is tolerated according to SIFT [30] and it has a GERP score of 4.47. Of the nine affected individuals that were included in the validation experiments, five carried the variant, while one unaffected family member was a carrier. The variant was absent from the unrelated control samples from the same region. The variant is found in the gnomAD browser at a low frequency (MAF 0.002), including one homozygous individual, and we note that the frequency is higher in the Finnish population (MAF 0.008). This does not exclude this as an interesting candidate gene, but we conclude that it is not a likely cause of intellectual disability considering the high population allele frequency.

Upon Sanger sequencing verification of the variant in *ACOT4*, additional variants were identified adjacent to the variants detected by exome analysis. In total, the variants made up a block with seven altered nucleotides changing the nucleotide sequence from CTCTAGCTTA to **A**TCT**TCAAAG** (bold letters indicate the identified SNVs), between the positions chr14(GRCh37):g.74060508–74,060,517. The variant segregates as a block substitution and the CADD PHRED score for the whole haplotype block was 22. The substitutions are predicted to change the amino acid sequence from ALAY to DLQS at the amino residues 187 to 190. At position 187 a non-polar amino acid (A, Alanine) was changed to an acidic (D, Aspartic acid). The A to Q (Glutamine) change at position 189 altered a non-polar amino acid to a polar, while the Y (Tyrosine) and S (Serine) at position 190 are both polar. The positions 187 and 189 are conserved from human to *Danio rerio* and the position has a GERP score of 5.25. We find that this variant is represented as three separate SNVs in gnomAD, with a frequency of around 15% in the Finnish population and therefore conclude that the block substitution variant in *ACOT4* is unlikely to be associated with disease.

### Rare variants outside of linkage peaks

To identify additional causative variants, the exome sequencing data was further analyzed without the filtering step requiring that at least four individuals should share the variant. This led to identification of a homozygous disease-causing variant at position chr6(GRCh37):g.74354306 G > A (rs80338794) in the *SLC17A5* gene in patients 829, 830 and 322. The mutation is known to cause Salla Disease.

Patient 301 is male and is the only affected individual in a branch of the pedigree, indicating that X-linked inheritance might play a role for this subject. Therefore, we screened this individual for rare variants on the X chromosome. The *FLNA* gene was the only gene that was found to contain a rare non-synonymous variant at position chrX(GRCh37):g.153591123 G > T (rs202109957), resulting in an amino acid alteration p.(Asn770Lys). Mutations in this gene cause Periventricular nodular heterotopia [31, 32], with ID as one of the clinical features. Since mutations Periventricular nodular heterotopia is an X-linked dominant disease we also screened other individuals for this variant. We found that individual 304, an affected female was heterozygous for the variant at position chrX(GRCh37):g. 153,591,123 G > T. The GERP score for the position is 4.44 suggesting that the region is conserved. We note that there are more than 20 hemizygous individuals for this variant in gnomAD, mainly from the Finnish population, indicating that this is likely a benign variant.

Moreover, we screened the male patient 3 for X-linked rare variants that could contribute to the phenotype, since he was the only one affected among his nine siblings. No obvious variant causing ID in this subject was identified. The only candidate disease variant we observed was a non-synonymous alteration in the *Collagen Type IV Alpha 6 Chain* (*COL4A6*) gene that is involved in X-linked non-syndromic sensorineural deafness, Alport syndrome and leiomyomatosis [33]. To our knowledge, mutations in the *COL4A6* has not been reported to cause ID however, some syndromic forms of hearing loss are associated with mental retardation too [34]. More research about this possible connection is called for.

We also screened the exome data to identify homozygous variants among ID patients, that were heterozygous or homozygous for the reference allele in individuals not affected by ID. The aim was to investigate whether additional genes, besides *SLC17A5* and the likely benign variants in *TPR*, *ACOT4 and FLNA,* could contribute to ID among some of the other affected subjects in the family. In total 148 SNVs were identified with homozygous non-synonymous changes or stop-gain effects. To select possible disease-causing variants among the 148 SNVs, we first searched for a shared variant among the three affected siblings 1, 2 and 319. However, no common SNV was identified. Next, we looked for possible disease causing SNVs in the rest of the affected family members. Identified variants were then compared to already known ID genes [3, 35] and we ended up with 8 possible disease causing variants. However, after manual inspection of the alignments to remove variants with low read coverage or variants that were located at the end of a read and likely to be false positive, no additional SNVs were found that could explain the disease phenotype.

## Discussion

Here we describe a large Swedish pedigree with several individuals affected by intellectual disability, and a smaller number of individuals affected by schizophrenia. The inheritance pattern and linkage results clearly indicate a complex inheritance.

### CNV results

The largest of the identified CNVs and potentially the most interesting, spans the gene *MAP3K4* and the last exon of the *AGPAT4* gene. It shows 84% overlap with a duplication previously found in a patient displaying symptoms such as attention deficit hyperactivity disorder and specific learning disability [36], although the pathogenicity of the CNV is uncertain. We further identified a deletion in patient 304 overlapping *KDM3B*, a gene involved in histone demethylation and chromatin remodeling. This gene was recently reported to harbor pathogenic variation associated with intellectual disability, short stature and facial dysmorphism [37].

### Linkage results

The linkage peak on 1q31-q41 coincides with the results obtained from an analysis of a large pedigree from Dagestan, where linkage to intellectual disability was identified to 1q41 [38]. Furthermore, linkage to 1q31 has also been found in a consanguineous family with members suffering from intellectual disability associated with ataxia [39]. The other three regions identified in the Swedish family have to our knowledge not been linked to intellectual disability.

### Exome analysis

Searching for variants shared by several patients, the exome analysis highlighted SNVs in the genes *TPR* and *ACOT4*. The *TPR* gene showed significant association with affected status in the family, while *ACOT4* did not reach statistical significance (*p* = 0.058). However, we initially found the *ACOT4* block substitution interesting due to its impact on the protein, and the fact that it was not reported in variant databases at the time.

The variant in *TPR* is rare, but with a previously reported frequency of 0.002 in the gnomAD browser, with a slightly higher frequency in the Finnish population. The individuals in the family in whom we identified the SNV were all heterozygous. *TPR* encodes a protein that attaches to the inner surface of nuclear pore complexes (NPCs) and is required for the nuclear export of mRNAs and proteins to the cytoplasm [40]. NPCs do not only play a role in nuclear transport, but also perform functions essential for normal cell growth and differentiation [41]. For example, it was recently shown that changes in NPC composition were required for myogenesis and neuronal differentiation in mice. Moreover, NPCs also appear to affect genome architecture and gene expression as reviewed previously [42]. The finding is interesting since another nuclear pore associated protein (NPAP1) is believed contribute to Prader-Willi syndrome [43] of which one of the major criteria is developmental delay/intellectual disability [44].

The block substitution in *ACOT4* is not represented as a variant in the gnomAD browser, and lead to three amino acid changes in the ACOT4 protein (Table 3), changing the protein sequence ALAY to DLQS at amino acid positions 187–190. The substitution is however reported as several independent SNVs, all with a frequency of around 15% in the Finnish population. The isoform has also been described in the UniProtKB database as a “Sequence conflict”. Based on the high population frequency of this variant in the Finnish population, we conclude that it is most likely a benign substitution.

### Heterogeneity within the family material

If variants in *TPR* and *ACOT4* are associated with ID in the family it appears as if the variants have reduced penetrance, since unaffected individuals are also carriers. Furthermore, we studied the exome data in more detail and an already known disease-causing variant in the *SLC17A5* gene was identified in patients 829, 830 and also 322. Homozygous or compound heterozygous mutations in *SLC17A5* cause Salla disease, first discovered in the village of Salla in Northern Finland where the founders of the Swedish family originate. The disorder is an autosomal recessive sialic acid storage disease where the patients are unable to transport sialic acid from the lysosomes. The disease causes a decrease in the myelin surrounding the neurons in the brain leading physical impairment and intellectual disability [45].

Patients 301 and 304 were hemizygous and heterozygous respectively for a rare variant in the *FLNA,* a gene involved in X-linked Periventricular nodular heterotopia, a disease where neurons do not migrate properly during the early development [31]. The condition is inherited in a dominant manner and most affected individuals with this disorder are females. Affected males usually die in utero, however there are some cases where hemizygous males have survived depending on the location of the mutation [46]. These two affected family members (301 and 304) were the only individuals with the rare variant. The position chrX(GRCh37):g. 153,591,123 has three reported alleles where the T-allele is silent and is reported to be benign. The A-allele identified in this study is a missense variant with low frequency (0.0006), similar to the frequency of the pathogenic variant in *SLC17A5*. If the gene is disease causing it is unlikely that the variant origins from a common ancestor, otherwise it would have been transmitted through 15 ancestors without clinical manifestation.

In our analysis we performed a thorough screen for pathogenic variants using dominant, recessive and X-linked models. Since we do not have access to DNA for parents, we are unable to screen specifically for de novo mutations in this data. There are other limitations in our study that are related to the family material. Since the pedigree is from a genetically isolated region with a small founder population, there may be identity by descent also in individuals that seem unrelated to the family. There will also be an enrichment of alleles that are rare in other populations due to the population bottleneck. This may partially explain how many different disease-associated alleles have risen to high frequency in the family. There are also limitations in the clinical assessment of the patients, which was performed up to two decades ago and was performed with the assumption of a shared genetic disorder. It is likely that a more recent and thorough clinical assessment of the patients would have revealed distinct differences that could be linked to causative genetic variants.

## Conclusions

In conclusion, our data indicate that more than one gene harbors pathogenic variation associated with ID in the family material, with a known pathogenic variant in *SLC17A5,* and likely pathogenic CNVs overlapping *MAP3K4*/*AGPAT4* and *KDM3B.* Our results show that what one large pedigree from a genetically isolated and homogeneous population, with suspected shared genetic contribution to ID, is in fact genetically heterogeneous within the pedigree. This may be the results of increased presence of deleterious variants in a genetically isolated region with a small founder population.

## Supplementary information

**Additional file 1: Figure S1.** The figure shows the karyotype of two individuals from the family. The karyotype on the left shows the chromosomes for an affected individual (individual 3) and the karyotype to the right shows the chromosomes for the unaffected brother (37177).

## Data Availability

The variants that were detected in the exome sequencing analysis and validated with Sanger sequencing were submitted to ClinVar (http://www.clinvar.com), as specified above. Accession numbers for the variants submitted to ClinVar are VCV000488642 – VCV000488646. The datasets analyzed during the current study are available from the corresponding author on reasonable request.

## Abbreviations

CADD: Combined Annotation Dependent Depletion score
CNV: Copy Number Variation
CT: Computed tomography scan
ID: Intellectual Disability
LD: Linkage Disequilibrium
NPC: Nuclear Pore Complex
SNP HiTLink: SNP High Throughput Linkage analysis system
SNV: Single Nucleotide Polymorphism
