# Supplementary material for: Linkage and exome analysis implicate multiple genes in non-syndromic intellectual disability in a large Swedish family
Source: BMC Med Genomics. 2019 Nov 6;12:156. doi: 10.1186/s12920-019-0606-4 (PMC6833288; doi:10.1186/s12920-019-0606-4)
Supplement: Supplementary file 1 — Additional file 1: Figure S1. The figure shows the karyotype of two individuals from the family. The karyotype on the left shows the chromosomes for an affected individual (individual 3) and the karyotype to the right shows the chromosomes for the unaffected brother (37177). [file 12920_2019_606_MOESM1_ESM.pdf]

S1 Fig. The figure shows the karyotype of two individuals from the family.

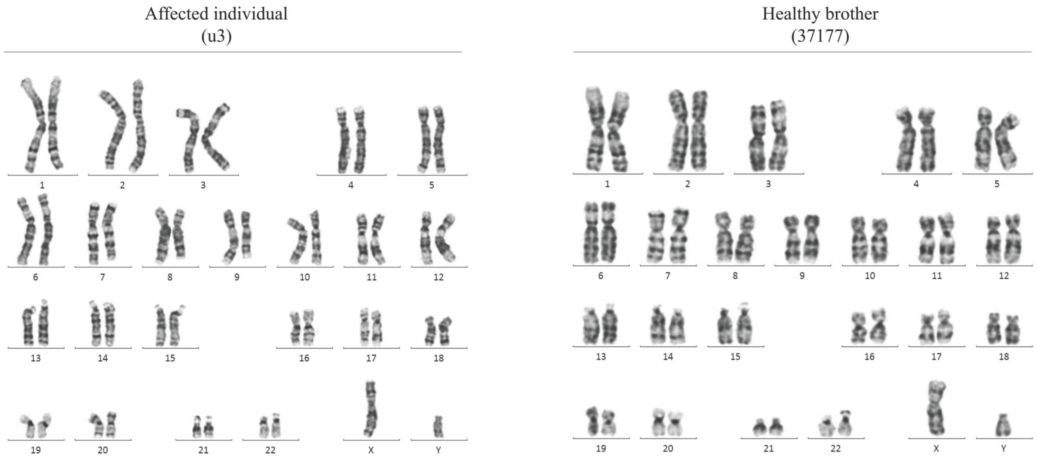

The karyotype on the left shows the chromosomes for an affected individual (individual 3) and the karyotype to the right shows the chromosomes for the healthy brother (37177).
